# Chronic Inflammation, Fibrotic Remodeling, and Regenerative Failure in Duchenne Muscular Dystrophy: Mechanisms and Therapeutic Opportunities

**DOI:** 10.3390/biomedicines14102192

**Published:** 2026-09-28

**Authors:** Jie Shi, Jiao Yang

**Affiliations:** 1 Department of Blood Transfusion, The Second Hospital of Lanzhou University, Lanzhou 730030, China; jieshi@lzu.edu.cn; 2 Department of Reproductive Medicine, The First Affiliated Hospital of Xi’an Jiaotong University, No. 277 West Yanta Road, Xi’an 710061, China

**Keywords:** Duchenne muscular dystrophy, fibro-adipogenic progenitors, chronic inflammation, fibrotic remodeling, muscle stem cells, regenerative failure

## Abstract

Duchenne muscular dystrophy (DMD) is an X-linked disorder initiated by dystrophin deficiency, but disease progression reflects more than sarcolemmal fragility. Recurrent myofiber injury sustains sterile inflammation through damage-associated innate immune signaling and downstream pathways including NF-κB and inflammasome activation. Persistent inflammation alters macrophage and fibro-adipogenic progenitor (FAP) behavior, promotes extracellular matrix remodeling, and creates a fibrotic niche that progressively limits effective repair. Regeneration is further compromised by both intrinsic muscle stem cell dysfunction and extrinsic constraints imposed by the remodeled microenvironment. This review integrates mechanistic and translational evidence linking these processes, with particular attention to FAP-centered stromal remodeling, immune–stem cell crosstalk, and tissue-specific differences between skeletal and cardiac muscle. We also evaluate emerging interventions targeting inflammatory priming, inflammasome activity, fibrotic remodeling, and regenerative competence while distinguishing established pathological mechanisms from predominantly preclinical therapeutic evidence. This perspective may help define stage-specific and niche-directed strategies that complement dystrophin restoration.

## 1. Introduction

Duchenne muscular dystrophy (DMD) is a severe X-linked neuromuscular disorder caused by mutations in the DMD gene, which result in the absence or near-complete loss of functional dystrophin [1,2,3]. As a key component of the dystrophin-associated glycoprotein complex, dystrophin stabilizes the sarcolemma during cycles of muscle contraction and relaxation [4,5,6]. Its deficiency renders myofibers highly vulnerable to contraction-induced mechanical stress, leading to membrane fragility, calcium overload, myofiber necrosis, and repeated cycles of degeneration and regeneration. Over time, this persistent injury results in progressive muscle wasting, loss of ambulation, respiratory compromise, and cardiomyopathy, which together define the major clinical burden of the disease. However, the pathobiology of DMD cannot be adequately explained by mechanical instability alone. Although dystrophin loss is the initiating molecular event, disease progression also involves chronic sterile inflammation, maladaptive stromal remodeling, extracellular matrix accumulation, and progressive regenerative failure [7]. In dystrophic muscle, repeated injury continuously releases danger-associated molecular patterns and other damage signals that activate innate immune pathways, sustain inflammatory cell infiltration, and reshape the tissue microenvironment. This persistent inflammatory state not only exacerbates myofiber damage but also promotes fibrotic remodeling and disrupts the regenerative machinery required for effective tissue repair [8,9,10,11]. Thus, DMD is a dynamic multicellular disease in which immune, stromal, and stem cell dysfunction contribute to irreversible muscle degeneration.

Several unresolved issues motivate a focused review of inflammation, fibrosis, and regenerative dysfunction in DMD. These processes are often analyzed separately despite evidence that their timing and cellular interactions influence disease progression [12,13,14]. In particular, FAPs can support acute repair but become maladaptively persistent during chronic injury, whereas the relative contributions of MuSC-intrinsic defects and niche-derived constraints remain incompletely resolved [14,15]. Translation is further limited because many proposed targets, including inflammasome inhibition and niche modulation, remain supported predominantly by preclinical evidence [16,17]. According, this critical narrative review focuses on the cellular and molecular interfaces connecting persistent inflammation, stromal remodeling, and impaired regeneration. We examine innate immune amplification, FAP/ECM remodeling, MuSC dysfunction, immune–stromal–stem cell crosstalk, tissue-specific cardiac implications, and emerging therapeutic approaches. Particular attention is given to evidence strength and model limitations rather than treating all proposed mechanisms and interventions as equally established.

This critical narrative review examines how recurrent myofiber injury is translated into persistent inflammatory signaling, stromal remodeling, and progressive loss of regenerative competence in DMD. Particular emphasis is placed on macrophage–FAP–MuSC interactions, extracellular matrix remodeling, tissue-specific differences between skeletal and cardiac muscle, and the translational maturity of emerging therapeutic strategies. Mechanistic findings are interpreted according to the strength and limitations of the available experimental and human evidence. Relevant literature was identified through targeted searches of PubMed and Web of Science, with priority given to original mechanistic studies, preclinical intervention studies, and clinically relevant evidence. Throughout this review, evidence is interpreted according to its experimental context. Mechanistic findings derived from murine models or ex vivo systems are described as preclinical evidence, observations in patient-derived cells or biopsies are considered supportive human evidence, and therapeutic efficacy is regarded as clinically validated only when demonstrated in interventional studies in patients. Where human validation remains limited, this uncertainty is stated explicitly.

## 2. From Dystrophin Deficiency to Inflammatory Amplification

Dystrophin deficiency initiates DMD pathology by destabilizing the sarcolemmal–cytoskeletal interface, but progressive tissue damage cannot be explained by mechanical fragility alone. Repeated membrane disruption, abnormal Ca^2+^ handling, oxidative stress, and myofiber necrosis continuously generate signals that activate innate immunity. Rather than resolving after individual episodes of injury, these signals accumulate within dystrophic muscle and progressively establish a state of persistent inflammatory priming. This section therefore considers inflammation as a continuum extending from primary myofiber injury and damage-associated molecular pattern (DAMP) release to TLR4/NF-κB-dependent innate immune amplification and, subsequently, inflammasome activation, pyroptotic signaling, and mitochondrial stress.

### 2.1. Myofiber Injury and DAMP Release

Dystrophin forms a structural and signaling link between the subsarcolemmal cytoskeleton, the dystrophin-associated protein complex, and the extracellular matrix. Its absence substantially increases the susceptibility of myofibers to contraction-induced membrane damage, particularly during lengthening contractions, thereby initiating repeated cycles of sarcolemmal disruption and necrosis [18,19]. Mechanical injury is accompanied by abnormal Ca^2+^ influx and impaired intracellular Ca^2+^ homeostasis, which activate proteolytic pathways and increase the vulnerability of dystrophic fibers to further injury [20,21]. Mechanical stress can additionally stimulate NADPH oxidase-dependent reactive oxygen species (ROS) production, which enhances Ca^2+^ entry through stretch-sensitive pathways and creates a reciprocal Ca^2+^–ROS amplification loop [22,23]. Thus, sarcolemmal instability is not an isolated structural abnormality but the initiating event of a broader stress response involving ionic dysregulation, oxidative injury, organelle dysfunction, and myofiber death.

Necrotic and severely stressed myofibers release intracellular molecules that function as DAMPs, thereby converting tissue injury into innate immune activation. ATP and nucleic acids released from damaged dystrophic fibers are particularly relevant because they provide persistent danger signals in a tissue undergoing recurrent necrosis [24]. Extracellular ATP is not merely a marker of myofiber damage: dystrophic muscle displays altered purinergic signaling, including increased P2X7 receptor expression and responsiveness, linking extracellular ATP to Ca^2+^ dysregulation and inflammatory activation [25,26]. Consistent with this model, pharmacological blockade of extracellular ATP/P2X signaling in mdx mice decreases leukocyte and macrophage infiltration, reduces inflammatory and profibrotic mediators, and is associated with improved regenerative features [27]. These observations illustrate how products released by damaged muscle can directly reinforce inflammatory signaling rather than disappearing once the initial injury has occurred.

High-mobility group box 1 (HMGB1) provides another experimentally supported link between myofiber damage and innate immunity. HMGB1 is redistributed outside the nucleus in dystrophic muscle and can act as an endogenous ligand for Toll-like receptor 4 (TLR4). In mdx mice, inhibition of HMGB1 with glycyrrhizin improves diaphragm pathology, while genetic ablation of TLR4 reduces inflammatory gene expression, macrophage accumulation, fibrosis, and functional impairment [28]. Collectively, these findings support a model in which recurrent myofiber necrosis continually replenishes a DAMP-rich microenvironment. Unlike acute muscle injury, where danger signaling subsides after tissue repair, dystrophin-deficient muscle remains exposed to newly generated ATP, HMGB1, nucleic acids, and other stress-associated signals, preventing complete inflammatory resolution and establishing the conditions for chronic innate immune amplification [24,28].

### 2.2. TLR4/NF-κB-Dependent Innate Immune Amplification

Among the receptors that translate tissue damage into persistent inflammation, TLR4 is particularly relevant to DMD. TLR4 expression and responsiveness are increased in dystrophic muscle, and genetic TLR4 deficiency in mdx mice improves diaphragm force while reducing macrophage infiltration, inflammatory gene expression, and fibrosis [28]. These findings indicate that TLR4 signaling is not simply a consequence of tissue injury but contributes functionally to disease amplification. DAMPs released from damaged myofibers may therefore establish a feed-forward circuit in which ongoing tissue injury repeatedly activates TLR4-positive myeloid cells, which in turn produce inflammatory mediators that further compromise dystrophic muscle.

More recent work has expanded this model by showing that the inflammatory consequences of recurrent DAMP exposure may extend beyond transient receptor activation. Bone-marrow-derived macrophages from mdx mice display features of trained immunity, including transcriptional hyperresponsiveness together with metabolic and epigenetic remodeling [29]. Importantly, the hyperresponsive phenotype can persist after bone marrow transplantation and can be induced by exposure to dystrophic muscle extracts, while the associated functional and epigenetic changes are TLR4-dependent [29]. These findings suggest that recurrent muscle damage may influence myeloid precursors before they enter dystrophic tissue, creating an innate immune system that is already primed for exaggerated responses. Trained immunity therefore provides a mechanistic explanation for why inflammation may remain amplified even when the intensity of an individual injury episode fluctuates [29,30]. However, this concept is currently supported primarily by murine mechanistic studies, and its persistence, stage dependence, and reversibility in patients with DMD remain to be established.

NF-κB functions as a major downstream hub connecting TLR and cytokine signaling with inflammatory gene expression. Persistent IKK/NF-κB activation has been demonstrated in both inflammatory cells and regenerating muscle fibers in dystrophic muscle [31]. Genetic reduction of NF-κB activity or cell-specific disruption of IKKβ in mdx mice showed that NF-κB has complementary pathological functions in different cellular compartments: macrophage NF-κB promotes inflammation and myofiber necrosis, whereas myofiber-associated NF-κB signaling can constrain regenerative responses [31]. Pharmacological IKK inhibition likewise improved muscle pathology and function in mdx mice, supporting a causal rather than purely associative role for this pathway [31]. NF-κB signaling is also relevant beyond skeletal muscle; cardiomyocyte-specific NF-κB inhibition in mdx mice improved cardiac function and altered calcium-regulatory transcriptional programs, emphasizing the tissue-dependent consequences of this signaling network [32].

Macrophages are central participants in this inflammatory amplification but should not be viewed as fixed binary populations. Dystrophic muscle contains macrophage states with inflammatory, reparative, and mixed properties whose abundance and function change with disease stage and local signals [33,34]. Recruitment of inflammatory monocytes through the CCR2 axis contributes to diaphragm pathology in mdx mice, whereas CCR2 deficiency reduces accumulation of inflammatory monocyte-derived macrophages and improves histological and functional outcomes [35]. Thus, the pathological consequence of innate immune activation reflects not simply the presence of macrophages, but their recruitment history, activation state, and interaction with the dystrophic niche. Within this network, TLR4 and NF-κB provide an upstream signaling framework that converts repeated DAMP exposure into sustained cytokine and chemokine production while simultaneously providing the transcriptional priming required for downstream inflammasome activation.

### 2.3. NLRP3 Inflammasome, Pyroptosis, and Mitochondrial Stress

Persistent innate immune priming creates a permissive environment for activation of the NLRP3 inflammasome. Canonical NLRP3 signaling requires transcriptional priming, commonly mediated through NF-κB, followed by activation in response to cellular stress signals such as ionic disturbances, ROS accumulation, mitochondrial dysfunction, or other forms of intracellular damage [36]. Activated NLRP3 assembles with the adaptor ASC and caspase-1, promoting maturation of IL-1β and IL-18 and cleavage of gasdermin D (GSDMD). The N-terminal fragment of GSDMD forms membrane pores, enabling inflammatory cytokine release and, when sufficiently extensive, execution of pyroptotic cell death [36]. In DMD, this pathway is particularly relevant because dystrophic muscle simultaneously contains strong priming signals and persistent cellular stressors capable of sustaining inflammasome activity.

Direct experimental evidence supports involvement of this pathway in dystrophic muscle. In mdx mice, pharmacological inhibition of NLRP3 with MCC950 reduced inflammation, macrophage infiltration, oxidative stress, myonecrosis, and fibrosis and improved muscle force and resistance to fatigue [16]. MCC950 also reduced active caspase-1 and cleaved GSDMD, providing evidence that NLRP3-dependent pyroptotic signaling contributes to dystrophic muscle injury [16]. Importantly, anti-inflammatory and anti-pyroptotic effects were also reproduced in primary human DMD myotubes, providing human-cell support for the pathway, although this does not constitute clinical validation in patients [16]. Comparative analyses of mdx mice and DMDmdx rats further show that inflammasome-associated proteins and cytokine cleavage vary according to tissue, age, and experimental model, with differences between skeletal muscle and heart [37]. NLRP3 activation should therefore be regarded as a well-supported preclinical mechanism in DMD, with complementary evidence from primary human DMD myotubes, but not yet as a clinically validated disease-driving pathway. Its relative contribution may vary according to muscle type, disease stage, and experimental context.

Mitochondrial dysfunction provides an important connection between primary dystrophin deficiency and inflammasome activation. Excessive cytosolic Ca^2+^ can be transferred into mitochondria, while ROS generation, altered mitochondrial permeability, and impaired bioenergetics increase organelle stress in dystrophic fibers [22,38]. Experimental studies have demonstrated increased susceptibility of mdx mitochondria to Ca^2+^-dependent permeability transition and abnormalities in mitochondrial mass and oxidative capacity [38]. More recent work has further shown that limiting mitochondrial Ca^2+^ overload can reduce oxidative stress, tissue degeneration, and fibrosis in mdx muscle, supporting a functional contribution of mitochondrial Ca^2+^ dysregulation to disease progression [39]. Damaged mitochondria can consequently become persistent sources of ROS and other stress signals capable of reinforcing inflammatory pathways, including NLRP3 activation [10].

Mitophagy normally limits this process by selectively removing dysfunctional mitochondria before they accumulate and amplify inflammatory signaling. Evidence from dystrophic models indicates that mitochondrial quality control is altered in DMD, although the direction and magnitude of these changes can depend on disease stage, muscle type, and experimental context [10]. A preclinical mechanistic link between mitophagy and inflammasome regulation has been demonstrated in mdx mice, where experimental TRIM72 overexpression enhanced mitophagy, reduced mitochondrial ROS, suppressed NLRP3 accumulation and IL-1β/IL-18 maturation, and alleviated muscle inflammation [40]. The protective effect was diminished when autophagic flux was inhibited, supporting a functional relationship between mitochondrial clearance and inflammasome control [40]. Recent studies of the PINK1–PARKIN pathway further reinforce the importance of mitochondrial quality control in dystrophic muscle and indicate that mitophagy responses may become inefficient under additional physiological stress [41].

Taken together, these findings support a continuous inflammatory axis rather than three independent mechanisms. Dystrophin deficiency first increases susceptibility to myofiber injury, Ca^2+^ dysregulation, oxidative stress, and necrosis. Repeated DAMP release then sustains TLR4/NF-κB-dependent innate immune activation and may induce durable myeloid priming. NF-κB and tissue stress subsequently provide the priming and activating signals required for NLRP3 assembly, while mitochondrial dysfunction and defective quality control further reinforce inflammasome activity. NLRP3-dependent cytokine maturation and GSDMD-mediated pyroptotic signaling can then generate additional inflammatory and damage signals, establishing a feed-forward loop that links primary mechanical injury to chronic inflammatory amplification and prepares the dystrophic niche for subsequent fibrotic remodeling and regenerative failure [16,29,31,40]. Figure 1 summarizes the continuous inflammatory axis linking primary dystrophin-dependent myofiber injury to persistent innate immune amplification. DAMP–TLR4/NF-κB signaling and mitochondrial stress converge on NLRP3-dependent pyroptosis, creating a feed-forward loop that sustains chronic tissue injury and prepares the dystrophic niche for subsequent fibrotic remodeling and regenerative failure.

## 3. Stromal Remodeling and Regenerative Failure

Persistent inflammation in DMD progressively reshapes the cellular and extracellular environment in which muscle repair occurs. Fibrosis is therefore not simply the terminal accumulation of scar tissue after repeated myofiber loss, but an active pathological process that alters immune-cell behavior, stromal-cell fate, extracellular matrix (ECM) organization, and muscle stem cell (MuSC) function. Experimental studies increasingly support a model in which fibro-adipogenic progenitors (FAPs), macrophages, MuSCs, and matrix components form an interconnected regulatory network rather than acting as independent downstream consequences of dystrophin deficiency [12,15,42]. This distinction is important because regeneration in DMD is initially active, but repeated injury occurs within a progressively less permissive niche. Over time, transient repair responses are replaced by persistent stromal activation, fibro-fatty remodeling, altered immune–stromal communication, and impaired regenerative output [14,43]. The resulting failure of repair reflects both changes in the surrounding niche and abnormalities within the MuSC compartment itself.

### 3.1. FAP and ECM Remodeling as an Active Component of DMD Progression

FAPs are muscle-resident mesenchymal stromal cells that have a context-dependent role in skeletal muscle repair. In acute injury, they rapidly expand and provide trophic and extracellular-matrix signals that support myogenic progenitor differentiation, while their subsequent contraction helps restore tissue homeostasis [44]. Type 2 immune signals also participate in this physiological response: eosinophil-derived IL-4 and IL-13 stimulate FAP proliferation and supportive functions while restraining adipogenic differentiation in experimental muscle injury [45]. Accordingly, transient FAP activation is not intrinsically pathological. Indeed, excessive pharmacological suppression of FAP expansion during acute regeneration can impair satellite-cell expansion and myogenesis, illustrating that a limited stromal response is required for effective tissue repair [46]. The pathological problem in DMD is therefore not the presence of FAPs per se, but the failure to appropriately terminate or redirect their injury-induced activation.

Chronic dystrophic injury profoundly alters this normal FAP response. Single-cell analyses have shown that FAPs exist as dynamic cellular states rather than a homogeneous population, and that muscular dystrophy is associated with persistent expansion of states enriched for profibrotic programs [12]. In particular, VCAM1-expressing FAPs with a profibrotic transcriptional profile normally appear transiently after acute injury but persist in dystrophic muscle when inflammatory resolution is disrupted [12]. More recent single-cell RNA sequencing of human DMD-derived FAPs has further demonstrated transcriptionally distinct proliferative, regulatory, inflammatory, and myofibroblast-like populations, with DMD FAPs showing enhanced expression of genes involved in ECM organization and remodeling [47]. These human observations support the relevance of FAP heterogeneity beyond murine models, although the functional contribution of individual human FAP states remains less well established than their transcriptional signatures.

The pathogenic consequences of FAP persistence extend beyond simple collagen production. Single-cell profiling of dystrophic muscle has identified FAPs as a major cellular source not only of ECM proteins but also of matrix-regulatory molecules, including connective tissue growth factor-related programs, matrix metalloproteinases, tissue inhibitors of metalloproteinases, latent TGF-β-binding proteins, and lysyl oxidase family members [15]. Importantly, the abundance and transcriptional state of these populations differ between the highly fibrotic diaphragm and less fibrotic limb muscles in mdx-derived models, emphasizing that FAP behavior is strongly shaped by the local tissue environment [15]. Metabolic state can also alter their pathological potential; dystrophic FAPs exhibit altered mitochondrial metabolism associated with increased adipogenic propensity, and experimental metabolic reprogramming can modify their contribution to the dystrophic phenotype [48]. Thus, FAPs integrate inflammatory, metabolic, and mechanical information and translate these signals into distinct regenerative, fibrogenic, or adipogenic outputs.

Macrophage-derived signals are particularly important in determining whether FAP expansion resolves or becomes persistent. During successful repair, inflammatory macrophage-derived TNF can promote apoptosis of excess FAPs, thereby limiting prolonged stromal accumulation [49]. In chronically damaged mdx muscle, however, macrophages acquire a TGF-β-rich profibrotic program that protects FAPs from apoptosis and favors their differentiation toward matrix-producing states [49]. This macrophage–stromal interaction is reinforced by an LTBP4-dependent mechanism in which dystrophic Ly6C-positive macrophages produce elevated latent TGF-β1, while FAPs contribute enzymes that activate the latent cytokine and thereby amplify collagen production [42]. More recent evidence further identifies a TGF-β–RUNX2 pathway in FAPs as a mechanistic link between macrophage-derived inflammatory cues and fibrogenic transcription in muscular dystrophy [50]. Together, these findings indicate that the inflammation-to-fibrosis transition is mediated by specific intercellular circuits rather than by a nonspecific accumulation of stromal cells.

The ECM generated during this process is itself biologically active. Fibrinogen accumulation in dystrophic muscle can engage leukocyte Mac-1/αMβ2 integrin signaling, enhance inflammatory cytokine production, increase macrophage-derived TGF-β, and promote fibroblast collagen synthesis [51]. Genetic disruption of the fibrinogen–αMβ2 interaction reduces inflammatory signaling and improves dystrophic pathology in mdx mice, demonstrating that matrix components can actively regulate inflammation rather than serving solely as structural products of fibrosis [52]. Similarly, the provisional matrix proteoglycan versican accumulates in DMD muscle and mdx diaphragm; genetic reduction of versican decreases macrophage infiltration and improves diaphragm contractile function [53]. However, the same intervention has much weaker and transient effects in relatively nonfibrotic mdx hindlimb muscles and does not consistently reduce inflammation [54]. These contrasting results demonstrate that ECM–immune coupling is highly dependent on muscle type, age, and the extent of pre-existing fibrosis.

The functional importance of the remodeled stromal niche is particularly evident in the severe D2-mdx model. Juvenile D2-mdx muscle exhibits poorly resolved inflammation, excessive FAP accumulation, fibrosis, and marked impairment of regenerative myogenesis [14]. Importantly, FAPs isolated from juvenile D2-mdx muscle reduce the fusion efficiency of otherwise healthy satellite cells in co-culture, providing direct evidence that pathological stromal cells can impose regenerative dysfunction on competent myogenic cells [14]. The spontaneous improvement of inflammatory and FAP responses in older D2-mdx animals is accompanied by improved myogenesis, further supporting the reversibility and stage dependence of at least some niche-mediated defects [14]. These findings provide preclinical evidence that pathological FAP and ECM remodeling can contribute causally to impaired myogenesis in severe dystrophic muscle [14]. However, this conclusion is derived primarily from the D2-mdx model and co-culture experiments, and the magnitude and reversibility of this niche-mediated effect in human DMD remain incompletely defined.

### 3.2. Extrinsic and Intrinsic Determinants of MuSC Dysfunction

DMD muscle retains substantial regenerative activity during early disease, as demonstrated by the abundance of centrally nucleated regenerating fibers and repeated activation of satellite cells. However, regeneration becomes progressively unable to compensate for continuous myofiber destruction. This should not be interpreted simply as depletion of the stem-cell pool. Rather, MuSCs must operate under conditions of repeated activation, persistent inflammation, abnormal ECM composition, stromal expansion, and recurrent exposure to newly damaged dystrophin-deficient fibers [7,55]. The regenerative defect therefore emerges from the interaction between extrinsic environmental constraints and cell-intrinsic abnormalities.

Extrinsic niche dysfunction can alter multiple stages of the regenerative program. Persistent FAP accumulation and fibrosis modify the physical and molecular architecture surrounding MuSCs, while inflammatory cytokines and stromal-derived factors influence activation, differentiation, fusion, and self-renewal [14,42]. In severe dystrophic muscle, the ability of D2-mdx FAPs to suppress satellite-cell fusion provides direct experimental support for this model [14]. Matrix-associated inflammation can also directly interfere with myogenesis; removal of fibrinogen-driven inflammatory signaling in mdx mice releases satellite cells from inhibitory signals and enhances regeneration [52]. These findings indicate that MuSC dysfunction cannot be understood independently of the immune and stromal environments in which these cells reside.

At the same time, dystrophin deficiency can directly affect MuSC biology. Dystrophin is expressed in activated satellite cells and participates in the establishment of cell polarity through interactions with MARK2 and the polarity regulator PARD3 [56]. In mdx-derived MuSCs, dystrophin deficiency has been associated with impaired PARD3 localization, abnormal mitotic spindle orientation, reduced asymmetric division, and decreased generation of committed myogenic progenitors [56]. More recent in vivo analyses have confirmed defective MuSC polarity and reduced myogenic commitment following acute injury in mdx mice, strengthening the evidence that regenerative failure includes a cell-autonomous component [57]. These findings are mechanistically important because asymmetric division allows the simultaneous maintenance of the stem-cell pool and generation of progenitors capable of differentiating into new myofibers.

The functional significance of this polarity defect is supported by rescue experiments. Activation of an EGFR–AURKA pathway increases asymmetric division of dystrophin-deficient satellite cells, expands myogenic progenitor output, enhances regeneration, and improves muscle force in mdx mice [58]. Such experiments provide causal evidence that at least part of the intrinsic regenerative defect is reversible rather than representing inevitable terminal exhaustion. Nevertheless, intrinsic dysfunction should not be generalized into the claim that all dystrophic MuSCs become irreversibly incompetent. Transplantation studies have shown that satellite cells isolated from both young and aged mdx muscle can retain substantial regenerative capacity when placed in a more permissive environment [59]. This apparent discrepancy is informative: it suggests that the phenotype of dystrophic MuSCs depends on the assay, disease context, and balance between reversible niche-induced dysfunction and more persistent cell-autonomous abnormalities.

Human evidence also argues against a simple depletion model. Histopathological analysis of DMD patient biopsies across disease stages has shown a progressive decline in regenerative activity accompanied by impaired activation and expansion of satellite cells and features consistent with cellular senescence [43]. At the same time, patient-derived FAPs expand as disease progresses, linking reduced myogenic activity with increasing fibro-adipogenic remodeling [43]. These findings support the biological relevance of regenerative decline in human DMD, but they do not by themselves establish whether satellite-cell senescence is a primary driver, a consequence of repeated injury, or one component of broader niche failure. Thus, the most consistent interpretation is that regenerative failure results from a changing balance between intrinsic MuSC defects and progressively restrictive environmental cues, with the relative importance of each component varying across disease stage and muscle type.

Chronic inflammatory signaling further modifies this balance. Macrophages are required for normal regeneration, and broad macrophage depletion in mdx muscle worsens pathology by changing satellite-cell identity, promoting aberrant adipogenic conversion, and reducing maintenance of the MuSC pool [60]. Conversely, subsets of macrophages can provide regenerative support; in mdx mice, CD206-positive macrophages have been shown to promote muscle growth and increase satellite-cell numbers through a Klotho-dependent mechanism [61]. These findings argue against interpreting inflammation as uniformly anti-regenerative. The problem in DMD is instead the persistence and mistiming of particular immune states, which disrupts the normal temporal sequence of inflammatory clearance, progenitor expansion, differentiation, and niche resolution.

### 3.3. Immune–Stromal–Stem Cell Crosstalk in the Dystrophic Niche

The preceding findings support a multicellular model in which macrophages, FAPs, and MuSCs continuously modify one another’s state. In acute muscle injury, immune-cell recruitment and stromal activation are tightly coordinated: inflammatory signals promote debris clearance and progenitor activation, FAPs transiently support myogenesis, and later resolution mechanisms restrict excess inflammation and stromal expansion [44,45]. DMD repeatedly resets this repair program before resolution is complete. As a result, cell states that are transient during physiological repair can become persistent, producing a niche characterized by simultaneous inflammatory activation, FAP accumulation, ECM deposition, and ineffective regeneration [12,49].

Macrophage heterogeneity is central to this process. Recruitment of CCR2-dependent Ly6C-high inflammatory monocytes contributes to diaphragm inflammation, fibrosis, and functional impairment in mdx mice, whereas disruption of CCR2 signaling reduces this infiltrating population and transiently improves dystrophic pathology [35]. However, recent lineage-tracing and single-cell studies show that suppressing monocyte recruitment does not permanently normalize the immune niche. In CCR2-deficient dystrophic muscle, resident Ly6C-low macrophages can expand and acquire pathogenic features, partly in response to FAP-derived CSF-1 [62]. This finding illustrates why macrophage biology in DMD cannot be adequately represented by a simple M1/M2 dichotomy and why suppression of a single recruited population may trigger compensatory changes in resident immune compartments.

Bidirectional FAP–macrophage signaling is similarly context-dependent. Macrophage-derived TNF promotes removal of excess FAPs after acute injury, whereas sustained TGF-β1 signaling during chronic damage protects FAPs from apoptosis and promotes fibrogenic differentiation [49]. Human studies provide additional evidence for macrophage–FAP interactions: FAPs are found in close proximity to CD68-positive macrophages in DMD muscle, and cytokines released by differently activated macrophages exert opposing effects on adipogenic differentiation of human FAPs [63]. Although these in vitro polarization experiments should not be equated directly with in vivo macrophage states, they reinforce the principle that the inflammatory environment determines whether FAPs support regeneration or contribute to fibro-fatty replacement.

Importantly, MuSCs are not merely passive recipients of immune and stromal signals. A recent mechanistic study in a DMD mouse model identified the YY1–CCL5–CCR5 axis as one potential pathway through which MuSCs may influence macrophage recruitment and stromal remodeling [13]. Loss of the transcription factor YY1 in dystrophic MuSCs increases CCL5 expression and secretion, enhancing recruitment of CCR5-expressing macrophages [13]. The resulting macrophage-rich environment increases TGF-β1 signaling, reduces FAP apoptosis, promotes FAP accumulation, and aggravates fibrosis [13]. Pharmacological blockade of CCL5/CCR5 signaling with maraviroc attenuates macrophage and FAP accumulation, reduces fibrosis, and improves muscle performance in the experimental model [13]. This work provides a particularly clear example of a bidirectional network in which an intrinsic change in MuSCs reorganizes immune and stromal compartments, which then feed back to alter regenerative conditions. Such interactions also help reconcile apparently contradictory observations regarding inflammation and regeneration. Macrophages can simultaneously participate in myofiber damage, FAP clearance, stromal activation, and MuSC support depending on their origin, activation state, timing, and local signals [42,60,61]. FAPs can promote differentiation during acute repair yet inhibit myogenesis when they persist in a dystrophic state [14,44]. MuSCs themselves can exhibit intrinsic polarity defects while retaining considerable regenerative potential under favorable conditions [56,59]. Therefore, none of these populations can be assigned a uniformly pathogenic or reparative role.

Collectively, the available evidence supports a model in which regenerative failure in DMD emerges from the progressive stabilization of a maladaptive multicellular niche. Recurrent injury sustains macrophage activation and stromal expansion; macrophage-derived TGF-β and related signals maintain fibrogenic FAP states; FAPs and ECM components reinforce inflammatory signaling and restrict myogenesis; and dysfunctional MuSCs can further alter the immune–stromal environment through paracrine mechanisms such as CCL5/CCR5 signaling [13,42,49,62]. This network is dynamic and potentially reversible, but most causal evidence still derives from murine or ex vivo systems. Human single-cell analyses and biopsy studies confirm FAP heterogeneity, progressive fibro-adipogenic remodeling, and declining regenerative activity, yet direct longitudinal validation of specific immune–stromal–MuSC circuits in patients remains limited [43,47]. Accordingly, the most promising therapeutic implication is not indiscriminate suppression of inflammation or stromal cells, but restoration of the temporal and cellular coordination required for effective repair. Table 1 summarizes the major inflammatory and fibrotic mechanisms that drive disease progression in DMD, highlighting the transition from sterile injury and innate immune amplification to inflammasome activation, extracellular matrix remodeling, and tissue-specific pathological deterioration. Table 2 outlines the major evidence supporting regenerative failure in DMD, emphasizing that impaired repair arises from both intrinsic muscle stem cell dysfunction and progressive remodeling of the inflammatory-fibrotic niche. Figure 2 summarizes the continuous inflammatory axis linking dystrophin-dependent membrane instability to persistent innate immune activation in DMD. DAMP–TLR4/NF-κB signaling and mitochondrial stress converge on NLRP3-dependent pyroptosis, thereby establishing a feed-forward inflammatory loop that drives subsequent fibrotic remodeling and regenerative failure.

## 4. Extension Beyond Skeletal Muscle: Cardiac and Systemic Implications

### 4.1. Inflammasome Activity in Skeletal Muscle Versus Heart

Although skeletal muscle degeneration is the most recognized feature of DMD, the disease is not restricted to limb muscle pathology. Cardiac involvement is a major cause of morbidity and mortality, particularly as respiratory and supportive care have improved patient survival. Importantly, the inflammatory mechanisms operating in the heart cannot be assumed to be identical to those in skeletal muscle [64,65]. While both tissues are affected by dystrophin deficiency and chronic stress, they differ in mechanical demands, regenerative capacity, cellular composition, and microenvironmental responses. These differences have significant implications for how inflammation is initiated, sustained, and translated into tissue injury in each organ.

Recent work comparing inflammasome-related activity in dystrophic skeletal muscle and heart has highlighted this point [37]. Although inflammasome signaling appears relevant in both tissues, the degree, timing, and pathological consequences of inflammasome activation may not be fully overlapping. This suggests that inflammatory injury in DMD follows tissue-specific logic rather than a single uniform program. In skeletal muscle, inflammasome activity is closely linked to repeated degeneration-regeneration cycles and persistent niche disruption [66,67,68]. In the heart, where regenerative potential is far more limited, inflammasome activation may more directly contribute to cardiomyocyte dysfunction, maladaptive remodeling, and long-term contractile decline [69,70,71,72]. This distinction is important because it prevents an overly simplified model in which cardiac disease is viewed merely as a delayed extension of skeletal muscle pathology. Instead, the heart should be considered a parallel but distinct target of dystrophin-deficient inflammatory remodeling. Appreciating tissue-specific inflammatory mechanisms may therefore improve both mechanistic understanding and therapeutic design, particularly when evaluating interventions that target pathways such as NF-κB, NLRP3, or oxidative stress across multiple organ systems.

### 4.2. Immune Mechanisms in DMD Cardiomyopathy

DMD-associated cardiomyopathy is increasingly recognized as an inflammatory and multicellular disease rather than a purely mechanical consequence of dystrophin loss in cardiomyocytes [73,74,75,76]. Chronic stress in the dystrophic heart activates immune pathways, remodels stromal compartments, and contributes to progressive fibrosis, conduction abnormalities, and ventricular dysfunction. As in skeletal muscle, macrophages play a central role in shaping this pathological environment. However, recent studies suggest that their effects in the heart may extend beyond conventional inflammatory and fibrotic functions. Emerging evidence indicates that specific macrophage subpopulations can contribute to dysfunction of specialized cardiac microenvironments, including neuro-cardiac junctional structures [77]. This is a particularly important advance because it expands the concept of DMD cardiomyopathy beyond cardiomyocyte death and interstitial fibrosis. It suggests that immune cells may also impair cardiac performance by disrupting communication networks that are essential for coordinated cardiac regulation. Such findings reinforce the idea that immune-mediated pathology in DMD is not limited to diffuse inflammation, but may also involve selective targeting of functional tissue interfaces. This broader view of cardiac immune pathology has several implications. First, it strengthens the rationale for integrating cardiac biology into inflammation-centered models of DMD. Second, these findings raise the possibility that macrophage-directed interventions could influence cardiac processes beyond fibrosis, although this remains a preclinical therapeutic hypothesis. Third, it also supports studying the dystrophic heart as a tissue with its own inflammatory and remodeling characteristics. In this sense, DMD cardiomyopathy should be interpreted as part of the same inflammatory-fibrotic-regenerative continuum, albeit one adapted to a tissue with minimal regenerative reserve. Together, these findings indicate that skeletal and cardiac muscle share inflammatory and fibrotic mechanisms but differ substantially in regenerative capacity and tissue-specific responses. Therapeutic evaluation should therefore consider both shared targets and organ-specific effects.

## 5. Therapeutic Opportunities

Therapeutic strategies targeting secondary DMD pathology differ substantially in translational maturity. Some anti-inflammatory and remodeling interventions have progressed to randomized clinical evaluation, whereas more selective approaches directed at innate immune signaling, inflammasome activity, stromal remodeling, or regenerative competence remain largely preclinical. Accordingly, this section emphasizes therapeutic evidence rather than re-describing the underlying mechanisms, with particular attention to experimental model, functional outcome, human validation, and translational limitations. Dystrophin-restoring strategies are not discussed in detail because they address the primary genetic defect; instead, the interventions considered here are positioned mainly as approaches that may reduce secondary tissue damage or improve the environment in which dystrophin-restoring therapies must act.

### 5.1. Clinically Translated Anti-Inflammatory and Tissue-Remodeling Strategies

Glucocorticoids provide the clinical benchmark for secondary-pathology-directed treatment in DMD. Randomized controlled studies established that prednisone improves muscle strength and functional performance in boys with DMD, while subsequent studies showed that deflazacort provides broadly comparable functional benefit with a different adverse-effect profile [78,79,80]. These data provide direct evidence that modulation of secondary inflammatory pathology can alter clinical disease trajectories even without restoring dystrophin. However, long-term glucocorticoid exposure is constrained by adverse effects involving growth, weight, bone health, and metabolic function, creating a need for anti-inflammatory strategies with improved tolerability [80].

Vamorolone represents a more recent example of clinically translated anti-inflammatory treatment. In randomized studies involving young boys with DMD, vamorolone produced dose-related improvements in motor outcomes [81]. In the subsequent randomized controlled extension, motor improvements with 6 mg/kg/day were maintained through 48 weeks, while participants transitioning from prednisone to vamorolone showed improvement in linear growth and reversal of prednisone-associated suppression of bone-turnover biomarkers [82]. These findings are important because they demonstrate that modification of inflammatory treatment can preserve functional benefit while altering aspects of the corticosteroid safety profile. Nevertheless, vamorolone does not directly correct the dystrophin defect, and its long-term effects on fibrosis, cardiac involvement, and late-stage regenerative failure remain distinct questions from its established short-term motor efficacy [82].

Givinostat provides particularly relevant clinical evidence for targeting tissue remodeling. In an early clinical study of 20 ambulant boys with DMD receiving stable corticosteroid treatment, more than 12 months of givinostat therapy increased the fraction of muscle tissue in biopsy specimens while reducing fibrosis, tissue necrosis, and fatty replacement [83]. Although that study was not powered to establish functional benefit, it provided direct human histological evidence that pharmacological treatment can modify the dystrophic tissue environment [83]. This finding was extended in the phase 3 EPIDYS trial, which randomized 179 ambulant boys to givinostat or placebo for 72 weeks on a background of systemic corticosteroid therapy. In the prespecified primary analysis population, deterioration in the four-stair climb was significantly smaller with givinostat than with placebo, although gastrointestinal adverse events, particularly diarrhea and vomiting, were more frequent [84]. The clinical development of givinostat therefore provides proof that targeting downstream muscle remodeling can generate measurable functional benefit in addition to histological effects.

These clinical studies provide an important benchmark for interpreting the more selective strategies discussed below. A preclinical intervention should not be considered comparable to glucocorticoids, vamorolone, or givinostat simply because it improves histological markers in mdx mice. Evidence becomes more persuasive when pathological improvements are accompanied by functional outcomes, are reproducible in severe or complementary disease models, and are ultimately supported by human muscle or clinical data.

### 5.2. Targeted Inhibition of Innate Immune and Inflammasome Signaling

Targeting TLR4 currently represents target-validation evidence rather than a clinically developed DMD therapy. Genetic ablation of TLR4 in mdx mice improved diaphragm force generation, reduced macrophage infiltration and pro-inflammatory gene expression, and decreased fibrosis [28]. These results support the therapeutic relevance of upstream innate immune signaling, but the intervention was genetic rather than pharmacological and was performed in a murine model. Consequently, the available evidence does not yet establish an optimal drug, dose, treatment window, or safety profile for chronic TLR4 inhibition in patients with DMD [28]. This distinction is important because strong mechanistic target validation should not be presented as equivalent to therapeutic readiness.

More direct pharmacological evidence is available for NLRP3 inhibition. In four-week-old mdx mice treated orally with the selective NLRP3 inhibitor MCC950 for two months, treatment reduced inflammatory infiltration, oxidative stress, myonecrosis, and fibrosis and increased force generation and resistance to fatigue [16]. The same study demonstrated reduced caspase-1/GSDMD-related pyroptotic signaling and reproduced anti-inflammatory and anti-pyroptotic effects in primary myotubes from patients with DMD [16]. This combination of animal functional outcomes and human-cell validation makes MCC950 one of the stronger target-specific preclinical examples within the inflammation-focused DMD literature. However, no controlled clinical evidence currently demonstrates that NLRP3 inhibition improves outcomes in patients with DMD, and long-term systemic inflammasome inhibition requires dedicated safety assessment [16].

Mitochondrial quality-control interventions provide an indirect approach to the same inflammatory endpoint. AAV-mediated TRIM72 overexpression in mdx mice enhanced mitophagy, reduced NLRP3-associated inflammatory signaling, and alleviated skeletal-muscle inflammation [40]. The study supports the feasibility of modifying mitochondrial stress–inflammasome coupling, but its translational maturity is lower than that of MCC950 because the therapeutic effect was obtained through experimental gene overexpression and complementary cell-line studies rather than a clinically deployable pharmacological intervention [40]. Thus, MCC950 and TRIM72 should be interpreted as complementary proof-of-concept strategies with different levels of direct targetability rather than as established treatment options.

### 5.3. Targeting Fibrotic and Stromal Remodeling

Direct modulation of pathological stromal remodeling has produced encouraging but heterogeneous preclinical results. Nilotinib is one of the best-characterized FAP-directed examples. In chronically injured muscle, including mdx mice, nilotinib restored susceptibility of pathological FAPs to TNF-mediated apoptosis and reduced muscle fibrosis [49]. The study provided causal evidence that altering FAP persistence can modify established fibrotic remodeling. However, nilotinib is a pleiotropic kinase inhibitor, and the experimental findings do not establish that chronic systemic administration would selectively modify FAP biology or provide an acceptable risk–benefit profile in children with DMD [49].

ECM-directed studies further demonstrate that therapeutic effects can depend strongly on muscle context. Genetic reduction of versican in fibrotic mdx diaphragm decreased macrophage infiltration and improved diaphragm contractile function and spontaneous physical activity [53]. In contrast, versican haploinsufficiency in less-fibrotic mdx hindlimb muscles produced only transient functional improvement and did not clearly suppress inflammation [54]. These paired studies are particularly informative for translation because they show that the same ECM intervention can produce substantially different outcomes depending on the degree of fibrosis and the muscle examined [53,54]. They therefore argue against assuming that antifibrotic efficacy measured in one muscle compartment will generalize across skeletal muscles or to the dystrophic heart.

Other recent interventions have attempted to simultaneously modify inflammatory and fibrotic readouts. Treatment with the slow-release adiponectin analogue ALY688-SR during very early disease in D2-mdx mice reduced indices of mitochondrial redox stress, fibrosis, and fiber atrophy in the diaphragm [85]. However, the treatment was initiated between postnatal days 7 and 28, and inflammatory cytokine responses were not uniformly suppressed, making this study more appropriately interpreted as evidence for early-stage remodeling rather than established reversal of advanced fibrosis [85]. The age and treatment timing are particularly important because prophylactic benefit in juvenile dystrophic mice cannot be assumed to predict efficacy once extensive fibro-fatty replacement is present.

ARC-18 provides another recent pharmacological example. Oral ARC-18 administered to two-month-old mdx mice for 60 days improved several motor-performance measures while decreasing inflammatory mediators, α-SMA, fibronectin, and collagen I [86]. Mechanistic experiments linked these effects to ACLY degradation and reduced Smad2/3 acetylation [86]. The inclusion of functional as well as molecular and histological endpoints strengthens the preclinical evidence, but the findings currently derive from a single murine study with prophylactic treatment. Independent replication, pharmacokinetic characterization, long-term toxicity assessment, and testing in more severe dystrophic models will therefore be necessary before this strategy can be considered translationally mature [86].

Several additional compounds demonstrate that FAP fate is pharmacologically tractable but currently have weaker DMD-specific translational support. Azathioprine suppresses adipogenic differentiation of FAPs isolated from dystrophic mice through altered AKT/PPARγ signaling [87], whereas WNT7A and retinoic-acid signaling reduce pathological adipogenic conversion or enhance regenerative FAP behavior in experimental muscle-injury models [88,89]. Because the latter studies were not performed primarily in DMD disease models, they are best regarded as supporting proof-of-principle for FAP modulation rather than direct evidence of efficacy in DMD. This evidence hierarchy should remain explicit when such candidates are discussed.

### 5.4. Restoring Regenerative Competence and Pathological Niche Communication

Regeneration-directed therapy has increasingly moved from general pro-myogenic concepts toward experimentally defined interventions. Isolecanoric acid (ILA), a GSK-3β-inhibitory natural product, enhanced myogenic differentiation in mouse and human stem-cell systems and improved regeneration and functional performance in dystrophic mice [90]. Treatment also reduced inflammatory responses and fibrosis following muscle injury, providing evidence that regenerative benefit can coexist with improvement of the surrounding pathological niche [90]. However, ILA remains an early preclinical candidate, with pharmacokinetic, toxicological, dosing, and reproducibility data required before its translational relevance can be established.

Cell-intrinsic reinforcement of MuSC function represents a different strategy. Targeted induction of HMOX1 in Pax7-positive satellite cells in dystrophic mice reduced muscle damage and produced a more anti-inflammatory tissue profile under exercise stress [17]. This study provides genetic proof of concept that improving the stress resistance of the stem-cell compartment can alter dystrophic pathology. Its immediate therapeutic translation is nevertheless limited because inducible, satellite-cell-specific transgenic HMOX1 expression is not itself a clinically deployable intervention [17]. Future development would therefore require a feasible method of achieving appropriately controlled MuSC-directed HO-1 activity.

IGF2R blockade has produced comparatively strong regenerative proof-of-concept data. IGF2R expression was increased in skeletal muscle from patients with DMD and mdx mice, and administration of neutralizing anti-IGF2R antibodies to mdx mice increased regeneration, improved muscle force, and normalized aspects of capillary architecture [91]. The presence of the target abnormality in human DMD tissue strengthens biological relevance, while the antibody intervention establishes pharmacological tractability in vivo [91]. Nevertheless, efficacy and safety have not yet been established clinically, and systemic manipulation of IGF signaling will require careful evaluation because of its broader effects on growth and tissue homeostasis.

Recent work has also identified post-transcriptional targets capable of increasing regenerative output. miR-33a deficiency enhanced regeneration and attenuated degeneration and fibrosis in mdx mice, whereas local and systemic administration of anti-miR-33a/b oligonucleotides improved the dystrophic phenotype [92]. The same study showed target-gene modulation in myotubes generated from a DMD patient-derived induced pluripotent stem-cell model, providing complementary human-cell evidence [92]. Although these data remain preclinical, systemic oligonucleotide administration makes this approach experimentally closer to a drug-development pathway than strategies that require permanent genetic modification of satellite cells.

Therapeutic manipulation of intercellular niche communication is another emerging approach. In a recent dystrophic mouse study, pharmacological blockade of CCL5/CCR5 signaling with maraviroc reduced macrophage and FAP accumulation, decreased fibrosis, and improved treadmill and voluntary-running performance in the experimental model [13]. Because maraviroc directly interrupted a defined MuSC–macrophage–FAP circuit, this study provides a useful example of targeting pathological crosstalk rather than broadly suppressing an entire immune-cell population [13]. However, the evidence currently relies heavily on one mouse study, and the activity of this pathway, effective therapeutic window, and consequences of prolonged CCR5 inhibition remain to be established in patients with DMD.

A further regenerative strategy involves thyroid-stimulating hormone receptor signaling. Experimental activation of this pathway restored regenerative function of skeletal muscle stem cells and improved dystrophic pathology in a rat muscular-dystrophy model [93]. Together with IGF2R blockade, HO-1 reinforcement, and miR-33 inhibition, these findings show that regenerative competence is pharmacologically modifiable rather than an immutable consequence of advanced dystrophic disease [17,91,92]. However, none of these approaches yet has clinical efficacy data comparable with the evidence available for established anti-inflammatory or anti-remodeling therapies.

### 5.5. From Single-Target Efficacy to Stage-Informed Combination Therapy

The therapeutic evidence reviewed above spans markedly different levels of maturity. Prednisone, deflazacort, vamorolone, and givinostat have been evaluated directly in patients [78,80,82,84], whereas MCC950, nilotinib, ALY688-SR, ARC-18, HO-1 reinforcement, IGF2R blockade, miR-33 inhibition, and CCR5 blockade remain preclinical [13,16,17,49,85,86,91,92]. These categories should not be discussed as though they provide equivalent evidence. In particular, improvements in histology or inflammatory markers should be distinguished from reproducible improvements in muscle force, motor performance, or clinically validated functional endpoints.

The available studies also indicate that therapeutic timing is likely to matter. ALY688-SR was tested during very early disease in D2-mdx mice [85], whereas versican-directed effects differed between a fibrotic diaphragm and less-fibrotic hindlimb muscles [53,54]. MCC950 was initiated in young mdx mice before advanced fibro-fatty replacement had developed [16], and ARC-18 was similarly tested in a relatively early prophylactic paradigm [86]. These experimental designs support biological efficacy but leave open the clinically important question of whether the same interventions can reverse established pathology. Future preclinical studies should therefore distinguish prevention from reversal and should incorporate disease stages and muscle groups that more closely reproduce the intended clinical setting.

Combination therapy should consequently be designed around complementary evidence rather than simple target accumulation. The phase 3 givinostat trial provides a clinically relevant example of an adjunctive strategy because participants were already receiving systemic corticosteroids when givinostat was added [84]. By contrast, there is currently insufficient clinical evidence to conclude that combining NLRP3 inhibition, FAP-directed therapy, regenerative stimulation, or niche-targeted drugs with one another—or with dystrophin-restoring therapies—will necessarily produce additive benefit. Such combinations should first demonstrate non-overlapping activity, acceptable safety, and functional benefit in relevant models before clinical translation.

A practical translational framework is therefore to match therapeutic strategy to the dominant, potentially reversible component of pathology. Inflammatory-targeted approaches may be most informative when inflammatory activity remains high; stromal and ECM-directed interventions require evidence that fibrosis is still modifiable; and regeneration-directed therapy is unlikely to succeed if the surrounding tissue has undergone extensive irreversible fibro-fatty replacement. Ultimately, these interventions are most appropriately viewed as potential complements rather than substitutes for restoration of the primary dystrophin defect. Their value will depend on whether they preserve viable muscle, restrain pathological remodeling, or extend the regenerative window sufficiently to enhance durable functional benefit. Table 3 summarizes emerging therapeutic strategies in DMD, showing that interventions targeting inflammatory amplification, fibrotic remodeling, and regenerative dysfunction may provide complementary or synergistic benefits.

## 6. Challenges and Future Perspectives

### 6.1. Limitations of Current DMD Inflammation Models

Despite major advances in understanding DMD pathogenesis, important limitations remain in the experimental models used to study inflammation, fibrosis, and regenerative failure. The conventional mdx mouse has been invaluable for mechanistic work, but it only partially recapitulates the severity and chronicity of human disease [94,95,96]. Although mdx muscle displays repeated degeneration-regeneration cycles and clear inflammatory abnormalities, the overall phenotype in many limb muscles is milder than that observed in patients, and regenerative compensation can remain relatively robust for a prolonged period [97,98,99]. As a result, some pathological features may be underestimated, particularly those related to severe fibrosis, niche collapse, and late-stage regenerative insufficiency [57,95].

More severe models, such as D2-mdx mice, help address some of these limitations by displaying more pronounced inflammation, fibrotic remodeling, and myogenic defects [100,101]. However, these models also introduce added biological complexity, including strain-specific modifiers and altered baseline tissue responses, which can complicate interpretation [100,102]. Similarly, diaphragm-focused studies often better reflect advanced dystrophic remodeling, but they do not necessarily capture all aspects of limb muscle biology [100]. These differences underscore a broader challenge in DMD research: no single model fully represents the spatial, temporal, and clinical heterogeneity of the human disease. Another limitation is that many studies examine isolated time points or single tissues, making it difficult to reconstruct how inflammatory, fibrotic, and regenerative programs evolve over the course of disease. DMD is fundamentally dynamic, and the relevance of a given pathway may differ substantially between early compensatory stages and late-stage decompensation. Future work will therefore benefit from integrative study designs that compare multiple tissues, disease stages, and model systems rather than relying on a single pathological snapshot.

### 6.2. Need to Move Beyond Simple Dichotomies

A second major challenge is conceptual. Much of the historical literature has relied on simplified frameworks, such as viewing macrophages through an M1/M2 dichotomy or interpreting muscle repair as a binary balance between degeneration and regeneration. While these models have heuristic value, they are increasingly inadequate for describing the complexity of DMD [34,103]. In dystrophic tissue, immune cells exist across diverse activation states that vary with tissue context, injury history, metabolic cues, and interactions with stromal and regenerative compartments [34]. Regenerative activity also exists along a continuum: it may remain quantitatively detectable while becoming qualitatively ineffective.

Moving beyond such dichotomies is particularly important for understanding why pathology persists even when nominally “repair-associated” processes are present. For example, macrophages may express features associated with both inflammatory and reparative functions while still contributing to maladaptive remodeling [34,103]. Similarly, MuSC activation may remain robust while self-renewal, differentiation fidelity, or long-term regenerative output progressively decline [57,104]. Binary classification systems cannot easily capture these mixed or transitional states, and may therefore obscure the mechanisms through which DMD pathology becomes chronic and self-reinforcing. A more useful framework is to treat DMD as a disease of dysregulated cellular states and pathological intercellular coordination. This approach better accommodates the possibility that cells can retain partial physiological functions while participating in disease-promoting networks. It also aligns with emerging multi-omics data showing that inflammatory, stromal, and regenerative populations occupy complex state continua rather than discrete categories [105,106]. Adopting such a framework will be essential for developing more precise mechanistic models and more rational therapeutic interventions. This conceptual shift also requires a methodological shift. To understand DMD as a disease of dynamic cellular states rather than fixed cell categories, future studies must adopt single-cell, spatial, and longitudinal strategies capable of resolving how pathological cell states emerge, interact, and respond to therapy.

### 6.3. Single-Cell and Spatial Technologies: From Descriptive Atlases to Causal Niche Biology

Single-cell and spatial technologies provide opportunities to resolve cell states, spatial organization, and intercellular interactions that cannot be adequately captured by bulk analyses [105,106,107]. Traditional bulk transcriptomic, histological, and biochemical approaches have been highly informative, but they average signals across heterogeneous tissues and therefore obscure the cellular origins, transitional states, and spatial organization of dystrophic pathology. This limitation is especially important in DMD, where disease progression is driven not by one cell type alone, but by changing interactions among myofibers, fibro-adipogenic progenitors (FAPs), macrophages, MuSCs, endothelial cells, pericytes, Schwann cells, and extracellular matrix compartments [15,105,108]. Single-cell and single-nucleus approaches can resolve these populations into disease-associated states, while spatial transcriptomics and multiplex imaging can determine where these states emerge and how they interact within injured, fibrotic, adipogenic, and regenerating regions [105,107]. The most important contribution of these technologies will not be the generation of larger cell atlases, but the reconstruction of pathological cell-state transitions. For example, FAPs should no longer be treated as a uniform stromal population. In dystrophic muscle, FAPs may occupy transient pro-regenerative states, persistent fibrogenic states, adipogenic states, inflammatory-response states, or matrix-remodeling states [15,47,107]. Similarly, macrophages cannot be adequately interpreted using a simple M1/M2 dichotomy; they likely exist along continua shaped by DAMP exposure, TLR signaling, metabolic stress, phagocytic activity, cytokine production, and stromal crosstalk. MuSCs may also progress through distinct states of activation, differentiation, stress adaptation, impaired self-renewal, and exhaustion. A major future challenge is therefore to define which cellular states are adaptive, which are maladaptive, and which represent reversible therapeutic windows. Spatial resolution is equally critical because DMD pathology is organized in niches rather than evenly distributed across muscle. Regions of active necrosis, macrophage accumulation, FAP expansion, collagen deposition, adipogenic replacement, vascular remodeling, and attempted regeneration may coexist within the same muscle but reflect different biological stages. Spatial transcriptomics and high-parameter imaging could identify the cellular neighborhoods in which inflammatory signals are converted into fibrotic or adipogenic remodeling. In particular, mapping macrophage–FAP–MuSC interfaces may clarify how failed inflammatory resolution suppresses myogenesis and promotes fibro-fatty replacement [12,15]. Such spatially resolved analysis would also help distinguish mechanisms that dominate in limb muscle, diaphragm, and heart, which may differ substantially in regenerative capacity, mechanical stress, and fibrotic progression. However, the field should avoid treating single-cell technology as an end in itself. Descriptive datasets alone will not establish causality. Future studies should integrate single-cell and spatial omics with lineage tracing, perturbation experiments, organoid or ex vivo muscle platforms, and functional rescue studies. Candidate pathways identified by transcriptomic analysis, such as FAP fate regulators, macrophage-derived cytokines, Wnt signaling components, TGF-β-related matrix programs, or chemokine axes such as CCL5/CCR5, must be tested experimentally to determine whether they drive disease progression or simply mark pathological tissue states. This distinction is essential for therapeutic translation. A critical future direction is therefore to move from static cell catalogues toward dynamic and intervention-oriented models of DMD progression. Longitudinal profiling across age, disease stage, muscle type, and treatment condition will be needed to determine when inflammatory, fibrotic, adipogenic, and regenerative programs become irreversible. Such work could identify stage-specific biomarkers, reveal which patients are most likely to benefit from anti-inflammatory or FAP-directed therapies, and guide rational combinations with dystrophin-restoring approaches. In our view, the real impact of the single-cell and spatial revolution will be realized only when these technologies are used to define causal pathological circuits and actionable regenerative windows, rather than simply to describe cellular heterogeneity.

### 6.4. Translational Priorities and Therapeutic Windows

Future studies should determine which inflammatory, stromal, and regenerative abnormalities are causal, reversible, and stage-dependent. Longitudinal human studies and biomarker development will be essential for defining therapeutic windows and identifying patients in whom secondary niche pathology remains active despite dystrophin-directed treatment. Preclinical candidates should be prioritized when their effects are reproducible across relevant disease models and muscle groups and are accompanied by meaningful functional outcomes. These steps will be necessary before mechanism-based combination strategies can be rationally translated into clinical practice.

## 7. Conclusions

Dystrophin deficiency initiates DMD, but the progression from recurrent myofiber injury to irreversible tissue loss is strongly influenced by persistent immune activation, stromal remodeling, and declining regenerative competence. Current evidence highlights macrophages, FAPs, extracellular matrix remodeling, and MuSC dysfunction as important components of this secondary pathology, although their relative contributions vary across disease stages, muscle groups, and experimental models. Therapeutic strategies directed at these processes should therefore be viewed primarily as potential complements to dystrophin restoration rather than substitutes for correction of the primary genetic defect. Future progress will depend on stronger human validation, identification of stage-specific biomarkers and therapeutic windows, and experimental approaches capable of distinguishing causal disease mechanisms from secondary markers of advanced tissue damage.

## Figures and Tables

**Figure 1:**
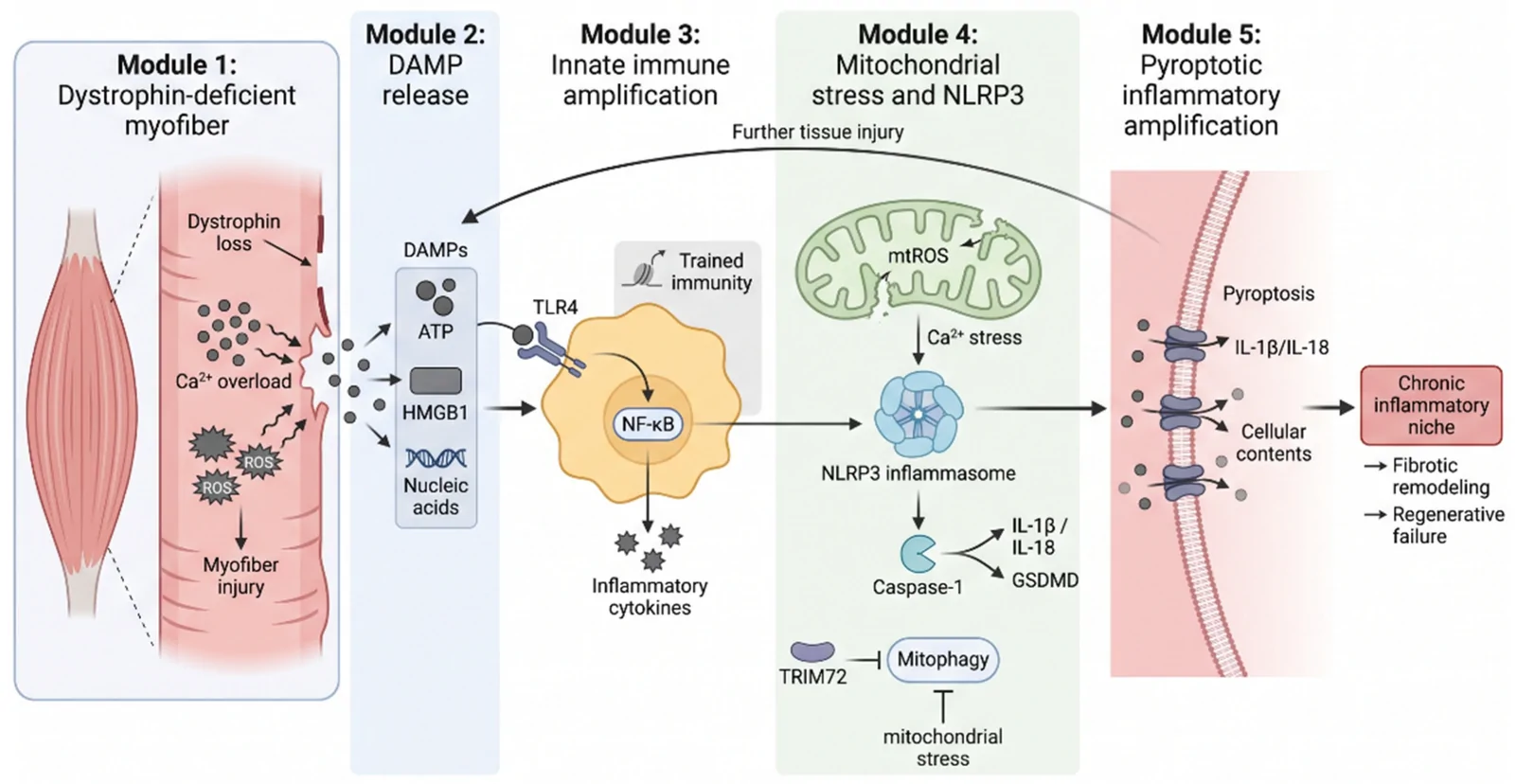
From dystrophin deficiency to chronic inflammatory amplification in DMD. Dystrophin loss promotes sarcolemmal injury, Ca^2+^/ROS stress, and DAMP release, which activate TLR4/NF-κB-dependent innate immune responses. NF-κB priming and mitochondrial stress converge on NLRP3, leading to caspase-1 activation, IL-1β/IL-18 maturation, and GSDMD-mediated pyroptosis. Pyroptotic injury further increases DAMP release, establishing a feed-forward inflammatory loop that promotes fibrotic remodeling and regenerative failure.

**Figure 2:**
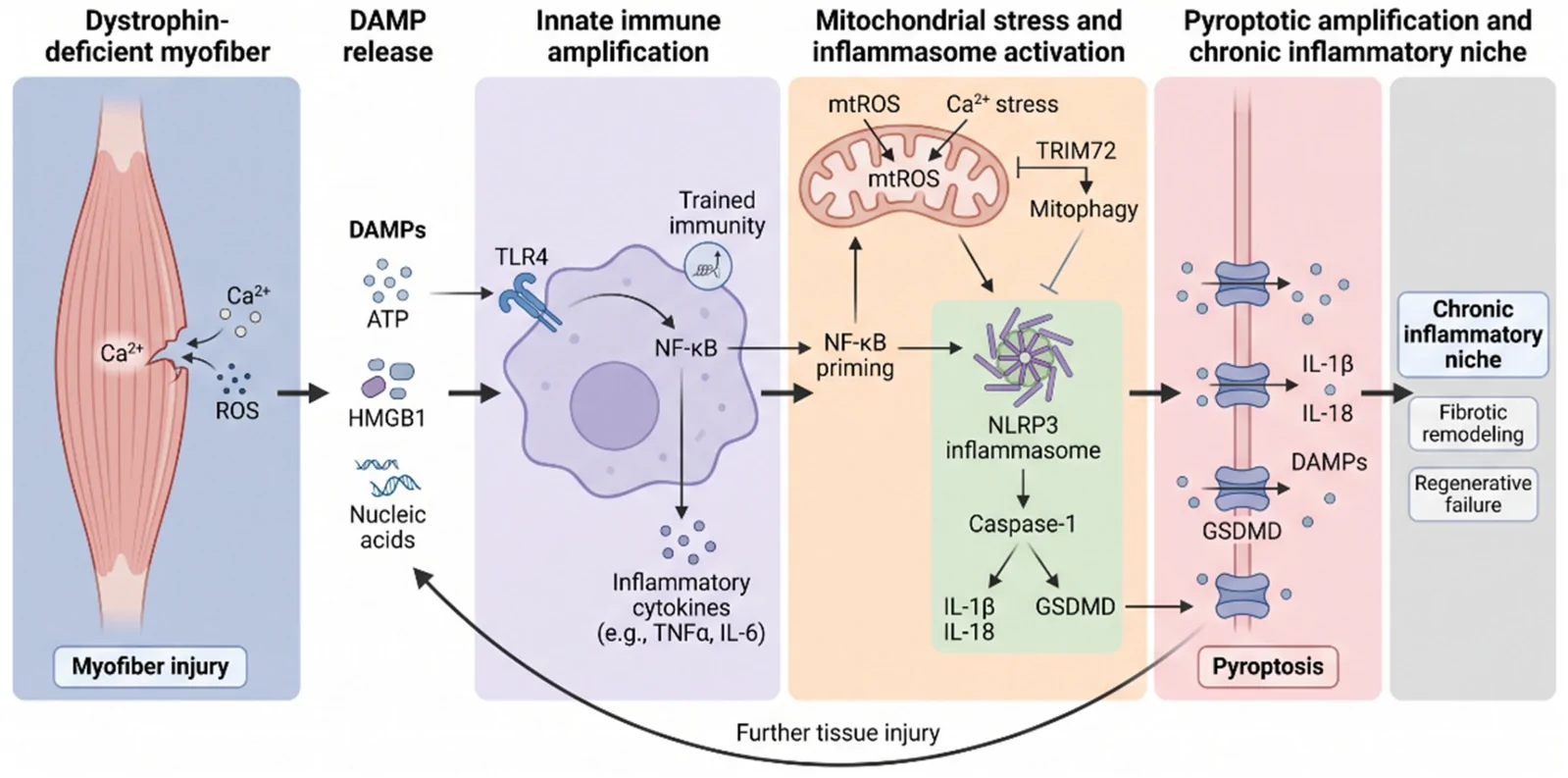
Dystrophin deficiency drives chronic inflammatory amplification in DMD. Loss of dystrophin promotes myofiber injury, Ca^2+^/ROS stress, and DAMP release, which activate TLR4/NF-κB-dependent innate immune signaling. NF-κB priming and mitochondrial stress converge on the NLRP3 inflammasome, leading to caspase-1 activation, IL-1β/IL-18 maturation, and GSDMD-mediated pyroptosis. Pyroptotic injury further enhances DAMP release, establishing a self-amplifying inflammatory loop that promotes fibrotic remodeling and regenerative failure.

**Table 1:** Key inflammatory and fibrotic mechanisms driving disease progression in Duchenne muscular dystrophy, with evidence strength and translational assessment.

Pathogenic Axis	Key Molecules/ Cells	Evidence Base/ Experimental Model	Main Mechanistic Finding	Evidence Strength	Translational Readiness	Key Limitations	PMID
Innate immune amplification	TLR4; myeloid cells; trained-immunity programs	mdx mice; bone-marrow-derived macrophages; bone-marrow transplantation/adoptive-transfer and in vitro muscle-extract experiments	TLR4-dependent metabolic and epigenetic reprogramming promotes persistent macrophage hyperresponsiveness and chronic inflammatory amplification.	Moderate (mechanistic preclinical)	Early preclinical; no clinically validated TLR4-directed strategy in DMD	Predominantly murine evidence; patient-level persistence, stage specificity, and therapeutic reversibility remain unestablished.	35169163
NF-κB-centered inflammatory signaling	IKK/NF-κB; p65; TNF-α; macrophages; myofibers	mdx mice with genetic and pharmacologic IKK/NF-κB perturbation; supportive observations in DMD tissue; mdx cardiac models	IKK/NF-κB activity in macrophages and myofibers promotes inflammation and necrosis and can constrain regeneration; cardiac NF-κB also alters calcium-regulatory programs.	Moderate (multi-model mechanistic evidence)	Preclinical/early translational; pathway is biologically validated but disease-modifying efficacy remains unproven	Pleiotropic pathway with cell- and tissue-specific effects; systemic inhibition may impair physiological immune and repair functions.	17380205; 30143619
Inflammasome activation and pyroptosis	NLRP3; caspase-1; IL-1β; IL-18; GSDMD	MCC950-treated mdx mice; primary human DMD myotubes; comparative mdx mouse and DMDmdx rat skeletal/cardiac tissue	NLRP3 activation promotes caspase-1/GSDMD-dependent inflammatory injury; MCC950 reduces pyroptosis and improves dystrophic phenotypes in mdx mice.	Moderate (interventional preclinical + human-cell support)	Preclinical; no clinical validation of NLRP3 inhibition in DMD	Human evidence is in vitro; rodent studies are small and show tissue-, age-, and model-dependent inflammasome activity.	36569900; 37239853
Mitochondrial stress-inflammation coupling	ROS; damaged mitochondria; mitophagy; TRIM72; NLRP3	AAV-mediated TRIM72 overexpression in mdx mice; complementary C2C12 perturbation experiments	Enhanced mitophagy can restrain mitochondrial stress-associated NLRP3 activation and reduce muscle inflammation.	Low-to-moderate (single-pathway mechanistic preclinical)	Early preclinical proof of concept	Relies on engineered overexpression and cell-line experiments; no direct human DMD validation or clinically feasible delivery strategy established.	36713032
Inflammation-to-fibrosis transition	Macrophages; stromal cells/FAPs; TGF-β-related and ECM-remodeling programs	Pharmacologic studies in mdx mice (growth hormone secretagogues, ARC-18) and early-stage D2.mdx mice (ALY688-SR)	Experimental modulation of inflammatory and profibrotic signaling alters ECM deposition, fibrosis-related readouts, and muscle function in dystrophic models.	Moderate but heterogeneous (preclinical intervention evidence)	Preclinical; candidate adjunctive strategies only	Different agents, models, ages, and endpoints limit direct comparison; some treatments were early/prophylactic, and human efficacy is unknown.	37122711; 38145301; 41054284
ECM–immune coupling	Versican; macrophages; ECM components	Versican haploinsufficiency in mdx-hdf mice; diaphragm and hindlimb muscle studies; observational evidence of versican accumulation in DMD muscle	Reducing versican decreases macrophage infiltration and improves diaphragm function, but effects are weaker and transient in less-fibrotic hindlimb muscle.	Moderate (mechanistic preclinical with human observational support)	Early preclinical; target concept not clinically validated	Genetic reduction is not equivalent to pharmacologic targeting; benefits are strongly muscle- and fibrosis-context dependent.	32632164; 39159389
Tissue-specific inflammatory remodeling	Skeletal muscle; diaphragm; heart; macrophage subsets; CSPG4	Age- and tissue-comparative studies in mdx mice and DMDmdx rats; mdx cardiac macrophage/CSPG4 perturbation study	Inflammasome and macrophage-associated remodeling differs across skeletal muscle and heart, supporting tissue- and stage-dependent inflammatory biology.	Moderate (multi-model preclinical)	Exploratory; mainly informs tissue- and stage-specific target selection	Evidence remains largely rodent-based; cross-tissue differences complicate generalization, and direct patient-level validation is limited.	37239853; 39523812

**Table 2:** Evidence for muscle stem cell dysfunction and pathological niche remodeling in Duchenne muscular dystrophy.

Study/Theme	Experimental Model	Main Finding	Interpretation for Regenerative Failure	Evidence Strength	Translational Readiness	Key Limitations	Representative Studies	PMID
Repeated degeneration-regeneration cycles become ineffective over time	mdx and D2-mdx dystrophic mouse models	Regenerative responses remain active, but repeated repair progressively fails to restore durable tissue integrity.	Supports regenerative failure as a combined consequence of intrinsic MuSC dysfunction and extrinsic niche constraints.	Moderate; convergent preclinical evidence	Pathophysiologic concept; not directly intervention-ready	Evidence is mainly murine. Timing, reversibility, and relative contributions of intrinsic versus niche mechanisms in patients remain uncertain.	Intrinsic MuSC dysfunction study; altered D2-mdx niche study	39723578; 37402716
Altered muscle niche contributes to myogenic deficit	D2-mdx mouse model of severe dystrophic disease	A pathological stromal/immune niche is associated with impaired myogenesis in severely dystrophic muscle.	Provides preclinical support for an extrinsic niche component of regenerative dysfunction.	Low-moderate; model-specific preclinical evidence	Early preclinical	D2-mdx represents severe disease and may not capture all DMD stages, muscles, or treatment contexts; direct human validation is limited.	Altered muscle niche contributes to myogenic deficit in the D2-mdx model of severe DMD	37402716
Intrinsic MuSC dysfunction	mdx mouse MuSCs	MuSCs exhibit cell-intrinsic abnormalities that compromise regenerative output.	Supports a cell-autonomous contribution to regenerative failure in addition to microenvironmental constraints.	Moderate; mechanistic preclinical evidence	Early preclinical	Cell-autonomous dysfunction is demonstrated primarily in mice; its magnitude, stage dependence, and interaction with the human DMD niche remain incompletely defined.	Intrinsic Muscle Stem Cell Dysfunction Contributes to Impaired Regeneration in the mdx Mouse	39723578
Impaired regenerative myogenesis in DMD MuSCs	mdx and D2-mdx models; DMD-associated MuSC molecular profiling	MuSCs show molecular impairments and altered cell-state/fate trajectories associated with reduced regenerative capacity.	Suggests qualitative stem-cell dysfunction rather than simple stem-cell depletion.	Moderate; multi-model molecular evidence	Discovery/biomarker stage	Profiling identifies disease-associated states but does not by itself establish causality; longitudinal human validation and functional rescue evidence remain limited.	Muscle stem cells in DMD exhibit molecular impairments and altered cell fate trajectories impacting regenerative capacity	40473604
Satellite cell-targeted cytoprotection	mdx; HMOX1Pax7Ind dystrophic mice	Targeted HO-1 expression in satellite cells attenuates dystrophic muscle pathology.	Provides proof of concept that increasing MuSC stress resilience can modify dystrophic pathology.	Moderate; genetic interventional preclinical evidence	Early preclinical proof of concept	Cell-specific genetic expression is not directly clinically deployable; durability, delivery, safety, and efficacy in human DMD are unknown.	Targeted expression of heme oxygenase-1 in satellite cells improves skeletal muscle pathology in dystrophic mice	38867250
MuSC-immune-stromal crosstalk (YY1-CCL5/CCR5 axis)	DMD mouse; MuSC-focused niche study	MuSC-derived CCL5 promotes CCR5-dependent macrophage recruitment and influences FAP-related fibrotic niche remodeling.	Supports MuSCs as active organizers of immune-stromal communication rather than passive responders.	Moderate; mechanistic mouse evidence	Early preclinical	Evidence relies heavily on a recent mouse study; pathway activity, cell specificity, therapeutic window, and safety have not been established in patients.	Skeletal muscle stem cells modulate niche function in DMD mouse through the YY1-CCL5 axis	39900599
Fibrosis as a barrier to regeneration	D2-mdx niche model; mdx diaphragm and hindlimb ECM/versican studies	ECM deposition and stromal expansion distort the regenerative niche; versican-related effects vary by muscle context.	Supports fibrosis and ECM remodeling as extrinsic constraints on repair while indicating tissue-specific heterogeneity.	Moderate; multi-model preclinical evidence	Exploratory preclinical	Findings vary across muscle groups and experimental contexts. ECM perturbation may also alter physiological repair; clinical benefit has not been demonstrated.	Altered D2-mdx niche study; versican diaphragm/hindlimb studies	37402716; 32632164; 39159389

**Table 3:** Emerging therapeutic strategies targeting inflammation, fibrosis, and regenerative failure in Duchenne muscular dystrophy, with evidence strength and translational assessment.

Intervention/ Strategy	Primary Target Axis	Evidence Base/ Experimental Model	Main Reported Effects	Evidence Strength	Translational Readiness	Key Limitations	PMID
TLR4/inflammatory priming modulation	Upstream innate immune amplification	Murine DMD/mdx model	TLR4 regulates trained-immunity-like inflammatory amplification; experimental modulation reduces excessive innate immune priming.	Moderate (mechanistic + interventional preclinical evidence)	Early preclinical	Evidence is predominantly murine; trained-immunity-like responses and optimal timing of TLR4 modulation remain insufficiently validated in patients with DMD.	35169163
MCC950	NLRP3 inflammasome/pyroptosis	DMD-model mice; DMD-related cellular studies	Reduces pyroptotic/inflammatory signaling and improves dystrophic muscle pathology.	Moderate (target-specific interventional preclinical evidence)	Early preclinical proof of concept	No clinical efficacy data in DMD; durability, systemic safety, optimal dosing, and effects on cardiac disease remain uncertain.	36569900
TRIM72-associated mitophagy restoration	Mitochondrial stress–inflammasome coupling	mdx mice	Promotes mitophagy, suppresses NLRP3 activation, and alleviates muscle inflammation.	Moderate (mechanistic preclinical evidence)	Early preclinical	Evidence is limited to animal studies; targetability, delivery strategy, dose–response relationships, and human relevance require validation.	36713032
Growth hormone secretagogues	Inflammation–fibrosis coupling	mdx mouse model	Modulate inflammatory responses and reduce fibrotic remodeling.	Low–moderate (single-model preclinical intervention evidence)	Exploratory preclinical	Findings derive from a murine model; mechanism specificity, durability, systemic endocrine effects, and clinical benefit in DMD are not established.	37122711
Isolecanoric acid	Inflammation + regenerative myogenesis	DMD-related muscle model	Improves inflammatory status, myogenic differentiation, and fibrotic readouts.	Low–moderate (preclinical proof-of-concept evidence)	Exploratory preclinical	Evidence is limited to experimental DMD-related muscle systems; pharmacokinetics, safety, reproducibility, and functional benefit in patients are unknown.	38159372
HO-1 targeted expression in satellite cells	MuSC protection/regenerative competence	Dystrophic mice; satellite-cell-targeted HMOX1 expression	Improves skeletal muscle pathology by strengthening satellite-cell-associated stress-protective pathways.	Moderate (genetic interventional preclinical evidence)	Early preclinical proof of concept	The cell-targeted genetic approach is not directly clinically deployable; translational delivery, durability, safety, and efficacy in human DMD remain unresolved.	38867250
Versican reduction/ECM modulation	ECM–immune coupling	mdx diaphragm and hindlimb muscle studies	Diaphragm: reduces inflammatory infiltration and improves function; hindlimb: transient functional benefit without clear reduction in inflammation.	Moderate (multi-study preclinical evidence with context-dependent effects)	Exploratory preclinical	Effects differ by muscle group and disease context, limiting generalization; ECM modulation may also affect physiological repair and has not been clinically validated.	32632164; 39159389
ALY688-SR	Diaphragm remodeling, redox stress, fibrosis	D2.mdx mice	Modifies early diaphragm remodeling and reduces mitochondrial/redox stress-related pathology.	Low–moderate (model-specific preclinical intervention evidence)	Exploratory preclinical	Evidence is confined to an early-stage severe mouse model; effects across later disease stages, other muscles, cardiac involvement, and patients are unknown.	38145301
CCR5 blockade-related approach	MuSC–macrophage–stromal crosstalk	Dystrophic mouse niche model/YY1–CCL5–CCR5 axis	Interrupts CCL5/CCR5-mediated pathological niche communication and improves muscle-related outcomes in the experimental model.	Moderate (mechanistic + interventional mouse evidence)	Early preclinical	Evidence relies heavily on a recent mouse study; pathway activity, therapeutic window, off-target immune effects, and efficacy in patients with DMD remain unestablished.	39900599
ARC-18	Inflammatory factors and fibrosis; ACLY–Smad2/3 acetylation	mdx mice	Improves motor performance and reduces inflammatory and fibrotic readouts.	Moderate (pharmacological interventional preclinical evidence)	Early preclinical	Evidence is limited to a murine model; target selectivity, pharmacology, long-term safety, reproducibility, and clinical efficacy require validation.	41054284

## Data Availability

No new data were created or analyzed in this study. Data sharing is not applicable to this article.
